# Metabolic phenotypes of doxorubicin-induced cardiotoxicity among patients with breast cancer

**DOI:** 10.1007/s11306-026-02469-7

**Published:** 2026-07-04

**Authors:** Amarnath Singh, Se-Ran Jun, Katherine Wallis, Renny S. Lan, Valentina Todorova, L. Joseph Su, Sam Makhoul, Ping-Ching Hsu

**Affiliations:** 1 Fay W. Boozman College of Public Health, University of Arkansas for Medical Sciences, 4301 W Markham St., #820, Rm 1224, Little Rock, AR USA; 2 Department of Biomedical Informatics, University of Arkansas for Medical Sciences, Little Rock, AR USA; 3 Winthrop P. Rockefeller Cancer Institute, University of Arkansas for Medical Sciences, Little Rock, AR USA; 4 Department of Pediatrics, College of Medicine, University of Arkansas for Medical Sciences, Little Rock, AR USA; 5 Department of Internal Medicine, University of Arkansas for Medical Sciences, Little Rock, AR USA; 6 Peter O’Donnell Jr. School of Public Health, UT Southwestern Medical Center, Dallas, TX USA; 7 CARTI Research Department, Little Rock, AR USA

**Keywords:** Breast cancer, Doxorubicin, Untargeted metabolomics, Cardiotoxicity

## Abstract

**Background:**

Doxorubicin (DOX)-based chemotherapy has improved survival outcomes in breast cancer patients but is often limited by doxorubicin-induced cardiotoxicity (DIC). Currently, no validated biomarkers can predict early DIC. Identifying novel biomarkers is essential for detecting patients at higher risk and enable timely interventions before irreversible cardiac injury occurs.

**Methods:**

Twenty-seven breast cancer patients treated with DOX-containing chemotherapy were stratified by change in left ventricular ejection fraction (LVEF): 19 patients who maintained normal cardiac function (normal, decline < 10%) and 8 who developed cardiotoxicity (abnormal, decline > 10%). Plasma samples were collected at baseline and after chemotherapy for untargeted metabolomic profiling. Both baseline and pre–post designs were employed to capture static and dynamic metabolic alterations associated with DIC. Stepwise logistic regression was used to filter non-informative metabolites, and predictive performance was further validated using Random Forest modeling.

**Results:**

A well-marked separation of plasma metabolomic profiles was observed between normal and abnormal cardiotoxicity groups at baseline (T0). Statistical analysis identified 100 significant metabolites at baseline (T0) and 78 metabolites after the first cycle of chemotherapy (T0-T1), with 10 metabolites common to both time-points: 3-phosphoglycerate, 2-hydroxyphenylacetate, inosine, taurine, suberate (C8-DC), sebacate (C10-DC), sphingadienine, oxindolylalanine. Machine learning models identified key metabolites (e.g., sebacate [C10-DC], 2-hydroxyhippurate, orotate, picolinate, and suberate [C8-DC]) as candidate predictors of cardiotoxicity, achieving moderate discriminatory performance in cross-validation, with higher specificity than sensitivity, indicating limited detection of abnormal cases.

**Conclusions:**

Metabolomic profiling shows potential for early detection of DIC in breast cancer patients, supporting personalized interventions to prevent irreversible cardiac damage.

**Supplementary Information:**

The online version contains supplementary material available at https://doi.org/10.1007/s11306-026-02469-7.

## Introduction

Breast cancer is the most diagnosed cancer and the leading cause of cancer death among women aged 20 to 49 years in the US (Xu et al., 2024). Doxorubicin (DOX) is a widely used anthracycline antibiotic for treating various cancers, but its application is often limited by its risk of causing congestive heart failure (CHF) (Belger et al., 2024). This cardiotoxicity arises from multiple mechanisms, including oxidative stress and mitochondrial dysfunction, leading to long-term cardiac damage (Palvia et al., 2024).

In 2020, there were approximately 2.3 million new cases of breast cancer globally (Arnold et al., 2022; Sung et al., 2021), with expectations to reach 4.4 million cases in 2070 (Soerjomataram & Bray, 2021). The increased incidence of breast cancer is mainly due to the increased prevalence of risk factors for breast cancer, such as obesity, physical inactivity, early menarche, shorter breastfeeding periods, and use of oral contraceptives (Britt et al., 2020; IARC, 2012; Zhang et al., 2020). Nonetheless, the mortality rate for breast cancer has steadily declined in recent years, largely due to advancements in early detection and the development of more effective therapeutic paradigms for breast cancer (Hendrick et al., 2021).

Doxorubicin (DOX), an anthracycline chemotherapy drug, remains one of the most potent and effective chemotherapy agents available and has been widely utilized in breast cancer treatment due to its mechanism of inhibiting topoisomerase II, preventing DNA replication and leading to cell death (Thorn et al., 2011). Although the number of long-term cancer survivors has increased because of DOX-based chemotherapy (Mattioli et al., 2004; Neppelenbroek et al., 2024), the number of patients experiencing DOX-induced cardiotoxicity (DIC) has also increased (Chatterjee et al., 2010; Gianni et al., 2007; Renu et al., 2018; Shakir & Rasul, 2009; Silber & Barber, 1995; Weiss, 1992; Zhang et al., 2009). A recent analysis of patients treated with DOX reported occurrence rates of 6% for clinically overt cardiotoxicity and 18% for subclinical cardiotoxicity (Lotrionte et al., 2013). Other studies reported cardiac dysfunction associated with DOX treatment varying from 30% in adult survivors to as much as 60% in children (Floyd et al., 2005; Swain et al., 2003). DOX-induced congestive heart failure is unpredictable and, once developed, carries a poor prognosis independent of oncological outcomes (Shakir & Rasul, 2009).

The most common clinical presentation of DIC begins with subclinical myocardial injury, followed by an early asymptomatic decline in the left ventricular ejection fraction (LVEF) that can progress to left ventricular dysfunction (LVD) and symptomatic heart failure (Floyd et al., 2005; Singal & Iliskovic, 1998; Yeh, 2006). Clinically, left ventricular function and diastolic function can be monitored by echocardiography and radionuclide angiography for patients receiving DOX treatment; however, echocardiography is operator dependent (Gottdiener et al., 2004), and there are risks associated with radiation exposure from radionuclide angiography (Volkova & Russell, 2011). Cardiac troponin T and troponin I have been used in non-clinical studies to assess early cardiac tissue damage (Hausner et al., 2013). However, their use as indicators of cardiotoxicity is limited and has not been validated in clinical studies (Herman et al., 1998; Monsuez, 2012; Yu et al., 2018). Thus, early biomarkers of DIC are critically needed, preferably through a minimally invasive method, allowing early intervention and patient stratification to identify patients at increased risk of DIC.

DIC involves a combination of metabolic and cellular pathways such as oxidative stress, mitochondrial dysfunction, apoptosis and ferroptosis (Bhutani et al., 2025; Hoeger et al., 2020). However, the relative contribution of each mechanism and their interactions remain unclear. Preclinical studies have shown some promising agents targeting mitochondrial functions (Chen et al. 2023a; Wang et al. 2024; Wu et al. 2022), but few have moved to human trials and long-term safety and efficacy are still under investigation.

In this study, we aimed to uncover potential metabolite biomarkers that could be used to identify early and subclinical indicators of DIC in patients with breast cancer. To do so, we examined the untargeted metabolomic profiles from a cohort of patients with breast cancer who developed an abnormal decline in LVEF > 10% or below 50% (Mookadam et al., 2014) and patients who maintained normal cardiac function.

## Materials and methods

### Study design

Patients with early-stage breast cancer eligible for DOX-containing chemotherapy were enrolled at the Winthrop P. Rockefeller Cancer Institute at University of Arkansas for Medical Sciences (UAMS). This study was approved by the Institutional Review Board (IRB) of UAMS (Protocol #130212), where all samples were processed and stored, and by the Central Veterans Healthcare System (CAVHS) IRB (Protocol #1423976-2), where aliquots of some samples were stored. All participants provided written informed consent, approved by the IRBs, permitting the use of their blood samples and medical records for research purposes. The inclusion criteria included patients 18–99 years of age with early-stage breast cancer (ER+/PR+/Her2−, ER+/PR−/Her2−, or triple-negative subtypes) at stages I to III. Participants were excluded if they were pregnant, breastfeeding, or had a prior history of chemotherapy or radiotherapy.

All patients were treated at the Winthrop Rockefeller Cancer Institute with a predefined protocol which included a combination of DOX (60 mg/m2) with cyclophosphamide (600 mg/m2) in each cycle for 4 cycles every 2 weeks. All participants signed an IRB approved informed consent in which they were informed in detail about the use of their blood samples and medical records for research purposes. Patients with pre-existing hypertension continued their antihypertensive medications (e.g., β-blockers and ACE inhibitors) during chemotherapy, and those with diabetes maintained their treatment with insulin or metformin concomitant with the DOX-based chemotherapy.

### Assessment of left ventricular ejection fraction as a measure of cardiac function

Cardiac toxicity was evaluated by clinical assessment of LVEF with Multigated Acquisition (MUGA) scan at baseline and after the fourth cycle of DOX-based chemotherapy. Patients were classified based on the magnitude of decline at the individual level rather than absolute decline of LVEF values in each group. A decline in LVEF of > 10% points from baseline or a post-treatment LVEF < 50% was considered indicative of cardiotoxicity and assigned to the abnormal group (ABN) (Belmonte et al., 2015; Zhang et al., 2012). Patients with an LVEF decline ≤ 10% points and post-treatment LVEF ≥ 50% were classified as normal group (NL).

### Sample collection processing

**Fig. 1:**
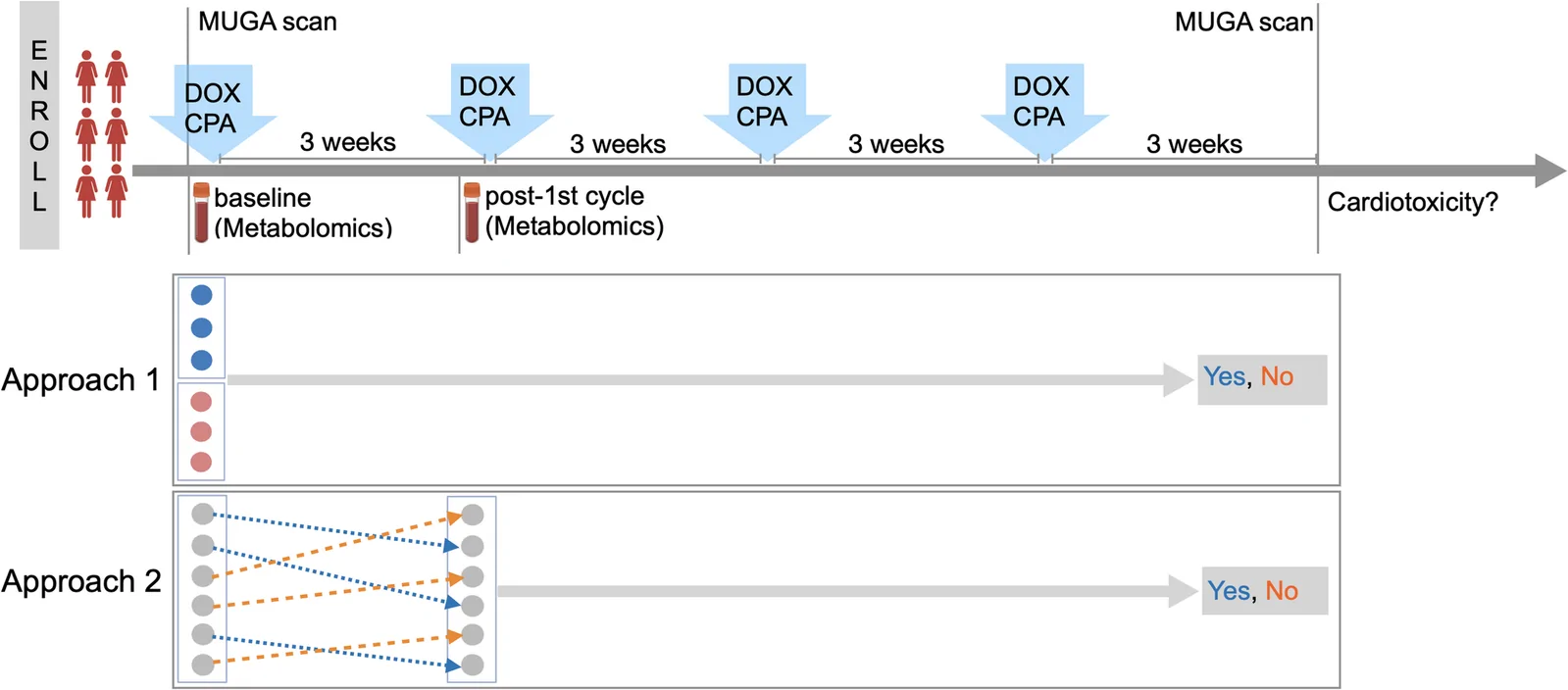
Study design and analytical approaches

Blood samples from each study participant were drawn during two time points: prior to chemotherapy (baseline, T0) and after the first cycle of chemotherapy (T1) (Fig. 1). Blood was collected in 6 mL BD Vacutainer EDTA tubes in the infusion clinic. Immediately after blood draw, the tube was inverted 8–10 times to mix the additive with the blood. Then the tube was placed immediately on ice. Tubes were then centrifuged at 3,000 rpm for 10 min at 4 °C within 2 h of blood collection. The plasma (supernatant) was collected in 1.5 mL cryotubes and stored at − 80 °C until shipment for metabolomic profiling. Samples remained frozen throughout storage and were shipped overnight on dry ice to Metabolon, Inc (Durham, NC, USA) for metabolomics profiling.

### Untargeted metabolite profiling

Upon receipt, samples were cataloged and instantly stored at − 80 °C until they were processed. On the extraction day, frozen samples were thawed on ice. Untargeted metabolomics profiling was performed with established protocols (Long et al., 2017) by Metabolon. The complete analytical process was monitored via quality-control steps and reference samples (Evans et al., 2009; Prakash, 2011; Sreekumar et al., 2009). Briefly, plasma samples were extracted and split into aliquots for analysis with a Waters ACQUITY ultra-performance liquid chromatography system and a Thermo Scientific Q-Exactive high-resolution/accurate mass spectrometer interfaced with a heated electrospray ionization (HESI-II) source and Orbitrap mass analyzer. Known metabolites were identified by automated comparison of ion features to a reference library of chemical standards followed by visual inspection for quality control (Dehaven et al., 2010). Samples were randomized across the platform run with quality-control samples spaced evenly among the injections. Metabolite abundance was estimated via area-under-the-curve for annotated peaks in the mass spectrogram (mass spectral counts) and was normalized for batch and run day in each cohort. Unknown metabolites were uniquely labeled in the format “X-12345”.

In this metabolomics study we included plasma sample collected from 27 patients at baseline and again after the 1 st cycle of chemotherapy enrolled between 2017 and 2020. A detailed description of the analytical protocol, metabolite identification, and normalization procedures is included in the Supplementary Methods.

### Mass spectrometry data processing and compound identification

Raw data were extracted, peaks were identified, and quality control was performed with Metabolon’s hardware and software. Compounds were identified by comparison to library entries of purified standards or recurrent unknown entities. Metabolon maintains a mass spectrometry (MS) library that contains the chromatographic retention time/index, mass-to-charge ratio (m/z), and MS/MS spectral data on 3,300 commercially available purified standard compounds. Metabolite identifications were based on 3 criteria: retention index within a narrow retention time/index window of the proposed identification, accurate mass match to the library ± 10 ppm, and the MS/MS forward and reverse scores between the experimental data and authentic standards. The MS/MS scores were based on a comparison of the ions present in the experimental spectrum to the ions present in the library spectrum. While there were similarities between these molecules based on one of these factors, the use of all 3 data points allowed us to distinguish and differentiate biochemicals. Metabolites were considered identified with high confidence when all 3 criteria were met. Additional mass spectral entries were created for structurally unnamed biochemicals, which were identified by virtue of their recurrent nature (both chromatographic and mass spectral). Although most annotations were based on standard compounds (Tier 1 identification), some were not. Compounds marked with an asterisk in Table S1 were not confirmed based on a standard but is highly probable based on the company’s confidence (Tier 2 identification).

A variety of curation procedures were carried out to ensure that a high-quality dataset was made available for statistical analysis and data interpretation (Herreros-Cabello et al., 2024; Ramsay et al., 2018; Rashidi et al., 2022). The quality control and curation processes were designed to ensure accurate and consistent identification of true chemical entities and to remove those representing system artifacts, mis-assignments, and background noise. Library matches for each compound were checked for each sample and corrected if necessary.

### Quantification and statistical analysis

Metabolite quantification and data normalization peaks were quantified via area-under-the-curve. A data normalization step was performed to correct variation resulting from instrument inter-day tuning differences. Essentially, each compound was corrected in run-day blocks by registering the medians to equal one and normalizing each data point proportionately.

### Data preprocessing

**Fig. 2:**
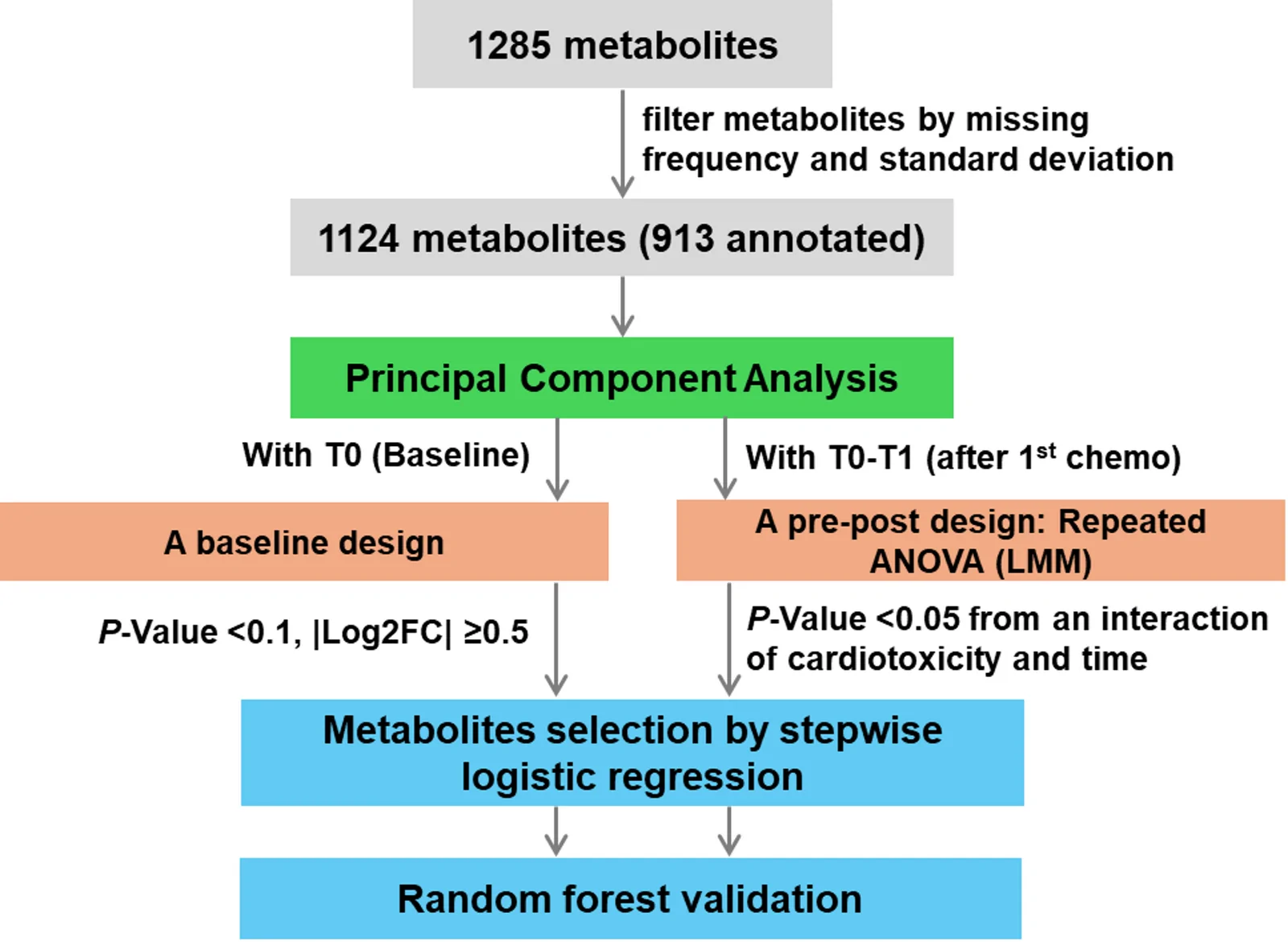
Analysis workflow for the untargeted metabolomic data

A total of 1,285 metabolites were detected from the plasma samples in this project. Prior to preprocessing, plasma volume was normalized with Metabolon’s in-house hardware and software. First, 156 metabolites with more than 50% missing values across samples were filtered out, followed by the removal of 5 metabolites with a standard deviation < 0.15. The values were log-transformed to improve normality, and missing values were imputed with the minimum value of the feature. The quality-controlled dataset, which contained 54 samples and 1,124 metabolites, was then used for downstream analyses (Fig. 2).

### Metabolomic data analysis

Principal component analysis (PCA) was first performed for exploratory visualization of global metabolomic patterns at baseline (T0) and after the 1 st cycle of chemotherapy. To identify potential metabolite biomarkers, we applied three distinct statistical modeling strategies: (1) screening of candidate metabolites using baseline and longitudinal pre–post analysis with linear mixed-effects modeling (LMM), (2) feature selection using stepwise logistic regression, and (3) exploratory validation using a Random Forest model (Figs. 1 and 2).

Further, for each approach, we used machine learning to select a reduced set of metabolites that could serve as early indicators of DIC in patients with breast cancer. For the univariate baseline approach, metabolites with a *P*-value < 0.1 from the t-test and an absolute |Log2FC| ≥ 0.5 were included in the initial screening set. Given the exploratory nature of this study and the limited sample size, this relatively lenient threshold was applied to avoid excluding potentially relevant metabolites. In the second approach, metabolites were selected based on a *P*-value < 0.05 for the interaction between cardiotoxicity and time variables in linear mixed-effects models (LMMs), adjusted for age, race, and BMI. These complementary approaches were designed to capture both baseline differences and dynamic treatment-related metabolic changes associated with DIC.

Metabolites identified from either screening approach were then carried forward to the feature selection stage. Stepwise logistic regression was applied to select a reduced set of metabolites associated with cardiotoxicity. We used the MASS R package to build a full logistic regression model, which incorporated all metabolites from the initial datasets; and then selected the most contributory metabolites through stepwise model selection based on the Akaike Information Criterion. Note that all samples were included in this analysis step, and analyses were restricted to annotated metabolites.

Finally, in the validation stage, Random Forest modeling was performed to evaluate the discriminatory ability of selected metabolites. Given the limited cohort size, leave-one-out cross-validation (LOOCV) was implemented to maximize data utilization, allowing each sample to serve once as a validation case while the remaining samples were used for model training. Model tuning was conducted by varying the mtry parameter, and performance was assessed using receiver operating characteristic (ROC) analysis, accuracy, and Cohen’s Kappa. Random Forest analyses were performed using the Random Forest and caret R packages.

### Statistical analysis

All statistical analyses were performed with *R*. Continuous variables are expressed as mean ± standard deviation (SD), and categorical variables are presented as n (%). Continuous variables were evaluated by two-sample t-tests, and chi-square (*X*^*2*^) tests were used to investigate the differences in distributions of categorical variables between 2 groups.

This figure outlines a multi-step process for analyzing metabolomic data. Initially, 1,285 metabolites were obtained from untargeted metabolomics assay, and then filtered based on missing frequency and standard deviation, resulting in 1,124 metabolites (913 annotated). Principal component analysis (PCA) was then performed to visualize for pattern recognition. Next, two separate statistical modeling strategies were applied. A baseline design: (1) Metabolites meeting a p-value < 0.1 and |Log2FC| > 0.5 were considered. (2) A pre-post (T0-T1) study design using repeated ANOVA (LMM), focusing on metabolites showing an interaction of cardiotoxicity and time with a p-value < 0.05. Metabolites showing an interaction of cardiotoxicity and time with a p-value < 0.05 were identified. From these significant metabolites, stepwise logistic regression was used to select informative markers. A Random Forest model further validated these metabolite markers to assess the robustness of the identified metabolite markers.

## Results

### Characteristics of study participants

In this study, 27 participants were enrolled and categorized into NL and ABN groups based on cardiac function assessments conducted at baseline and following the final cycle of chemotherapy. The mean age of study participants in the NL group was 51.0 years (SD = 12.7), and similarly, the mean age in the ABN group was 50.7 years (SD = 12.7). The average body-mass index (BMI) was 32.7 in the NL group and 32.2 in the ABN group (Table 1). No significant differences were observed between NL and ABN groups on hormone receptor status. Hypertension was similarly distributed between groups (NL: 47.4% vs. ABN: 50.0%), while diabetes was present only in the NL group (10.5% vs. 0%). However, these differences were not statistically significant. Notably, mean LVEF values were similar between groups at both baseline and post-treatment; however, classification into ABN was based on individual-level declines rather than group mean differences.

**Table 1:** Characteristics of patients included in this study

	^a^NL (*n* = 19)	^a^ABN (*n* = 8)	^b^ *P*
Age, Mean ± SD	51.0 ± 12.7	50.7 ± 12.7	*0.91*
BMI, Mean ± SD	32.7 ± 7.1	32.2 ± 7.3	*0.67*
Race, n (%)			*0.23*
European American	14 (73.7%)	4 (50.0%)	
African American	5 (26.3%)	4 (50.0%)	
Breast cancer, n (%)			*0.63*
ER+/PR+/Her2-	11 (57.9%)	6 (75.0%)	
ER+/PR-/Her2-	1 (5.3%)	0 (0.0%)	
ER-/PR-/Her2-	7 (36.8)	2 (25.0%)	
ER, n (%)			*0.55*
Positive	12 (63.1%)	6 (75%)	
Negative	7 (36.8%)	2 (25%)	
PR, n (%)			*0.70*
Positive	11 (57.9%)	4 (50%)	
Negative	8 (42.1%)	4 (50%)	
Triple-negative, n (%)			*0.55*
Yes	7 (36.8%)	2 (25%)	
No	12 (63.2%)	6 (75%)	
Hypertension, n (%)			*0.90*
Yes	9 (47.4%)	4 (50.0%)	
No	10 (52.6%)	4 (50.0%)	
Diabetes, n (%)			*0.34*
Yes	2 (10.5%)	0 (0.0%)	
No	17 (89.5%)	8 (100%)	
LVEF baseline (%), Mean ± SD	61.9 ± 7.3	63.0 ± 6.7	*0.02*
LVEF after 4 cycles (%), Mean ± SD	59.5 ± 7.0	59.9 ± 7.0	*0.05*

^a^ NL: patients with LVEF decline ≤10 percentage points from baseline and post-treatment LVEF ≥50%; ABN: patients with LVEF decline >10 percentage points from baseline or post-treatment LVEF <50%. ^b^ P-values represent differences between NL and ABN for the characteristics. Continuous variables were evaluated by two-sample t-tests, and chi square tests were used to investigate the differences in distributions of categorical variables between 2 groups.Abbreviations: NL, normal group; ABN, abnormal group; BMI, body-mass index; ER, estrogen receptor; PR, progesterone receptor; Her, human epidermal growth factor receptor; LVEF, left ventricular ejection fraction.

**Fig. 3:**
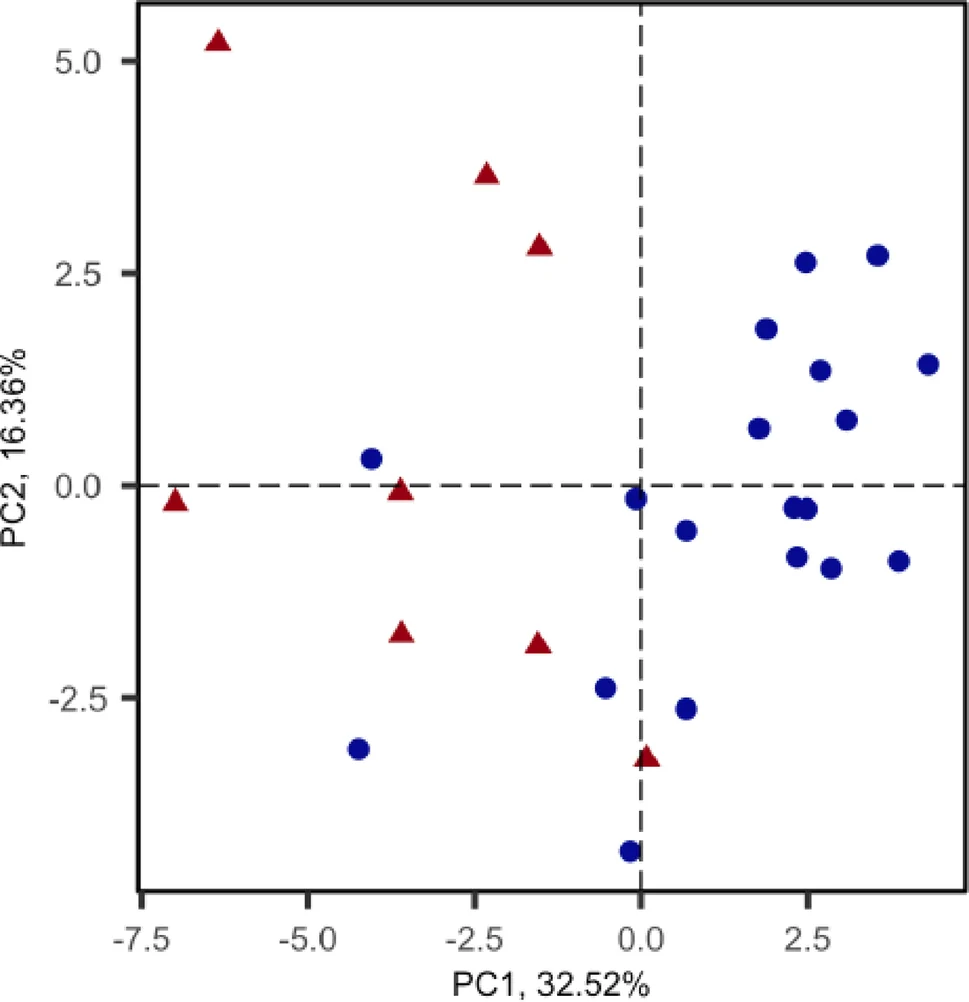
Principal component analysis of untargeted metabolomic profiles at baseline (T0). PCA plots show sample clustering by cardiotoxicity group (normal vs. abnormal ejection fraction) at baseline with 32.52% variance after data filtering out missing values < 50%, metabolites with standard deviation < 0.15, and after log transformation

### Metabolomic profiles of normal CVD versus abnormal groups at baseline and follow-up

#### At baseline

**Fig. 4:**
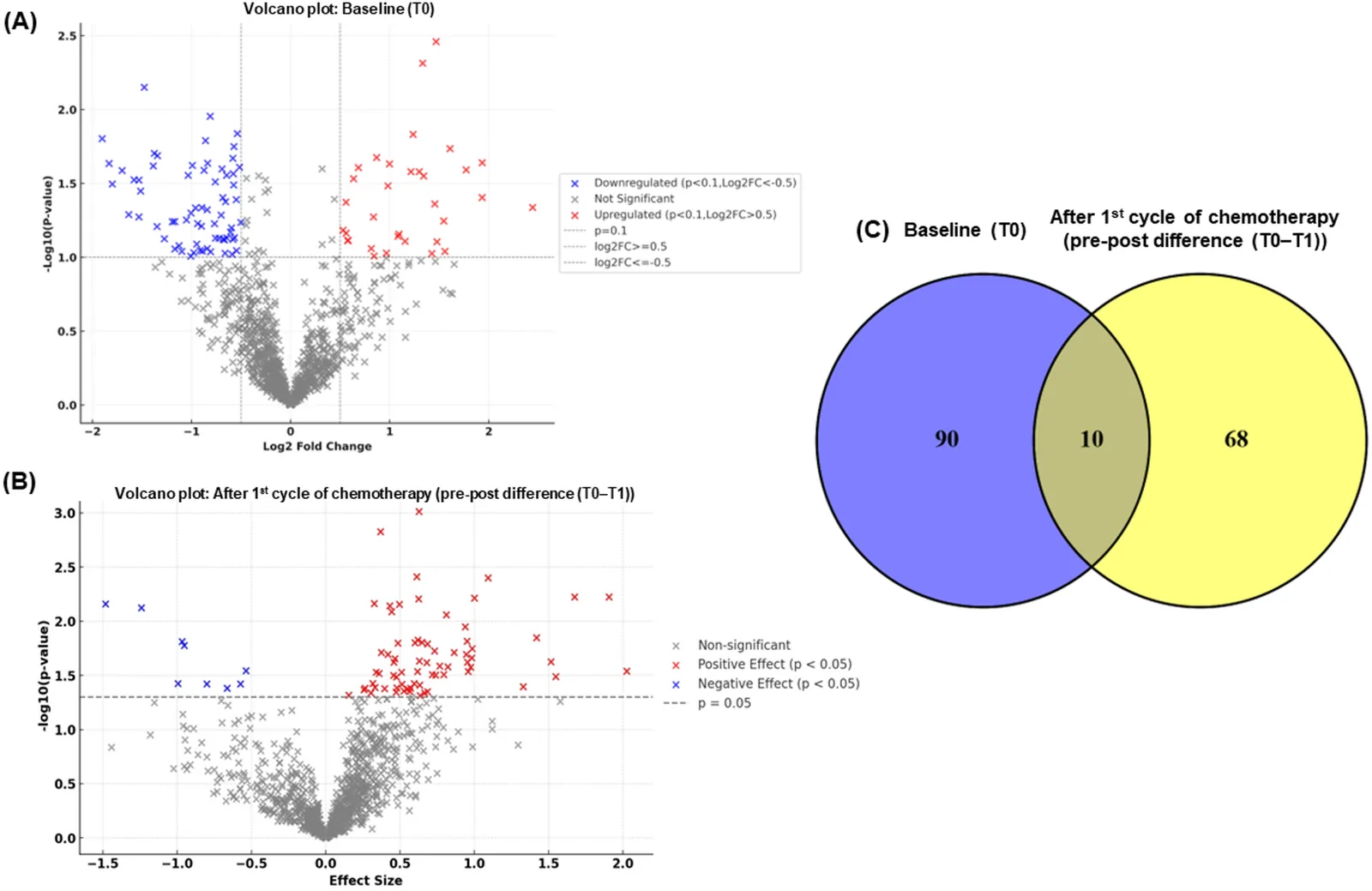
Differential plasma metabolites between NL vs. ABN at baseline and pre-post differences after the first cycle of DOX-containing chemotherapy depicted by volcano plots and Venn diagram. **(A)** Volcano plot of baseline metabolites, with the x-axis representing |Log2FC| ≥ 0.5 and the y-axis showing -log10 (*P*-value). Significant metabolites (*P*-value < 0.05) were highlighted as colored crosses, with downregulated metabolites in ABN group indicated by blue crosses and upregulated metabolites in ABN group indicated by red crosses. **(B)** Volcano plot of pre-post metabolite differences after the 1 st cycle of DOX-containing chemotherapy, with effect size on the x-axis and –log₁₀(P-value) on the y-axis. Significant metabolites (*P*-value < 0.05) were highlighted as colored crosses, with negative effect size metabolites represented by blue crosses (increased in the ABN group at T1) and positive effect size metabolites represented by red crosses (decreased in the ABN group at T1). (**C**) Venn diagram analysis between baseline and pre-post metabolite differences after the first cycle of DOX-containing chemotherapy

Principal component analysis revealed distinct clustering of metabolomic profiles between NL and ABN groups at baseline (Fig. 3). To identify baseline metabolite markers predictive of cardiac outcome, we applied a univariate analysis and selected top 100 metabolites showing significant differences at baseline between NL vs. ABN groups based on the cutoff of *P* < 0.1 and |Log2FC| ≥ 0.5 (Table 2 and Fig. 4A). Among these, 78 metabolites were annotated and 22 unannotated; 67 metabolites were downregulated and 33 were upregulated in ABN group. Notably, elevated levels of 3-sulfo-alanine, taurine, oxindolylalanine, suberate (C8-DC), sebacate (C10-DC), sphingosine, o-cresol sulfate, and 3-hydroxypyridine sulfate suggest alterations in amino acid, lipid, xenobiotic metabolic pathways. Decreased levels of 2-hydroxyhippurate, S-methylcysteine, 3-hydroxyisobutyrate, Indolepropionate, 1-palmitoyl-2-linoleoyl-GPE (16:0/18:2), 1-palmitoyl-2-oleoyl-GPE (16:0/18:1), 1-palmitoyl-2-palmitoleoyl-GPC (16:0/16:1)*, 1-palmitoyl-GPE (16:0) suggest alterations in amino acid and lipid metabolic pathways. Collectively, these findings highlight substantial metabolic disturbances in lipid and amino acid metabolism pathways in the ABN group, with implications for chemotherapy-associated cardiotoxicity and altered systemic metabolic regulation.

**Table 2:** Metabolite markers different by cardio function group (NL vs. ABN) at baseline identified by ANOVA with *P*-values < 0.1 and |Log2FC| > 0.5

Chem ID	Name	Sub Pathway	Super Pathway	*P*-value	Log2FC
382	3-sulfo-alanine	Methionine, Cysteine, SAM and Taurine Metabolism	Amino Acid	0.003	1.47
100,001,211	Sebacate (C10-DC)	Fatty Acid, Dicarboxylate	Lipid	0.005	1.33
100,001,870	1-palmitoyl-2-linoleoyl-GPE (16:0/18:2)	Phosphatidylethanolamine (PE)	Lipid	0.011	−0.81
498	Retinol (Vitamin A)	Vitamin A Metabolism	Cofactors and Vitamins	0.015	−0.54
1105	Alpha-tocopherol	Tocopherol Metabolism	Cofactors and Vitamins	0.016	−1.90
823	Pyruvate	Glycolysis, Gluconeogenesis, and Pyruvate Metabolism	Carbohydrate	0.016	−0.86
100,020,487	N-acetyl-isoputreanine	Polyamine Metabolism	Amino Acid	0.018	−0.57
100,020,545	N2-acetyl, N6,N6-dimethyllysine	Lysine Metabolism	Amino Acid	0.020	−1.38
501	Salicylate	Drug - Topical Agents	Xenobiotics	0.021	−1.35
100,000,016	Suberate (C8-DC)	Fatty Acid, Dicarboxylate	Lipid	0.021	0.87
100,001,567	1-palmitoyl-GPE (16:0)	Lysophospholipid	Lipid	0.021	−0.58
100,020,822	Dihydrocaffeate sulfate (2)	Food Component/Plant	Xenobiotics	0.023	1.93
100,009,078	1-oleoyl-2-linoleoyl-GPE (18:1/18:2)*	Phosphatidylethanolamine (PE)	Lipid	0.023	−0.84
100,001,658	Taurolithocholate 3-sulfate	Secondary Bile Acid Metabolism	Lipid	0.023	−1.83
1869	2-hydroxyhippurate (salicylurate)	Benzoate Metabolism	Xenobiotics	0.024	−1.39
100,015,839	Dihomo-linoleoylcarnitine (C20:2)*	Fatty Acid Metabolism (Acyl Carnitine, Polyunsaturated)	Lipid	0.025	−0.52
100,009,066	1-palmitoyl-2-oleoyl-GPI (16:0/18:1)*	Phosphatidylinositol (PI)	Lipid	0.025	−0.69
361	Inosine	Purine Metabolism, (Hypo)Xanthine/Inosine containing	Nucleotide	0.026	1.77
100,008,991	1-palmitoyl-2-docosahexaenoyl-GPE (16:0/22:6)*	Phosphatidylethanolamine (PE)	Lipid	0.026	−0.87
100,001,806	O-cresol sulfate	Benzoate Metabolism	Xenobiotics	0.026	1.30
100,008,976	1-stearoyl-2-linoleoyl-GPE (18:0/18:2)*	Phosphatidylethanolamine (PE)	Lipid	0.027	−0.58
100,009,181	1-stearoyl-2-oleoyl-GPI (18:0/18:1)*	Phosphatidylinositol (PI)	Lipid	0.028	−0.65
100,020,851	3-ethylcatechol sulfate (1)	Food Component/Plant	Xenobiotics	0.028	1.34
100,022,120	Oxindolylalanine	Tryptophan Metabolism	Amino Acid	0.029	0.63
100,022,006	Bilirubin degradation product, C17H18N2O4 (2)**	Partially Characterized Molecules	Partially Characterized Molecules	0.030	−1.58
100,006,642	Glycodeoxycholate 3-sulfate	Secondary Bile Acid Metabolism	Lipid	0.030	−1.53
100,004,541	Acisoga	Polyamine Metabolism	Amino Acid	0.031	−0.76
1090	Bilirubin	Hemoglobin and Porphyrin Metabolism	Cofactors and Vitamins	0.032	−1.80
100,015,835	Cerotoylcarnitine (C26)*	Fatty Acid Metabolism (Acyl Carnitine, Long-Chain Saturated)	Lipid	0.032	−0.58
100,001,083	Indolepropionate	Tryptophan Metabolism	Amino Acid	0.036	−1.51
100,020,536	4-ethylcatechol sulfate	Benzoate Metabolism	Xenobiotics	0.039	1.93
100,015,840	Dihomo-linolenoylcarnitine (C20:3n3 or 6)*	Fatty Acid Metabolism (Acyl Carnitine, Polyunsaturated)	Lipid	0.039	−0.68
100,015,836	Ximenoylcarnitine (C26:1)*	Fatty Acid Metabolism (Acyl Carnitine, Monounsaturated)	Lipid	0.041	−0.55
100,002,458	3-methylglutaconate	Leucine, Isoleucine, and Valine Metabolism	Amino Acid	0.042	−0.66
100,004,112	3-methyl catechol sulfate (1)	Benzoate Metabolism	Xenobiotics	0.044	1.45
100,006,098	3-hydroxypyridine sulfate	Chemical	Xenobiotics	0.046	2.44
1025	Pipecolate	Lysine Metabolism	Amino Acid	0.046	−0.90
212	5-methylthioadenosine (MTA)	Polyamine Metabolism	Amino Acid	0.046	−0.96
2054	Ethylmalonate	Leucine, Isoleucine, and Valine Metabolism	Amino Acid	0.048	−0.84
100,006,644	Taurodeoxycholic acid 3-sulfate	Secondary Bile Acid Metabolism	Lipid	0.050	−1.01
100,001,657	Glycolithocholate sulfate*	Secondary Bile Acid Metabolism	Lipid	0.053	−1.53
512	Taurine	Methionine, Cysteine, SAM, and Taurine Metabolism	Amino Acid	0.053	0.84
100,020,546	N2-acetyl, N6-methyllysine	Lysine Metabolism	Amino Acid	0.056	−1.05
100,006,438	Citraconate/glutaconate	TCA Cycle	Energy	0.057	1.55
100,000,436	Glycodeoxycholate	Secondary Bile Acid Metabolism	Lipid	0.057	−1.17
100,022,007	Bilirubin degradation product, C17H18N2O4 (3)**	Partially Characterized Molecules	Partially Characterized Molecules	0.057	−1.19
1528	1-palmitoyl-2-linoleoyl-GPI (16:0/18:2)	Phosphatidylinositol (PI)	Lipid	0.058	−0.50
100,022,004	Bilirubin degradation product, C16H18N2O5 (2)**	Partially Characterized Molecules	Partially Characterized Molecules	0.059	−0.94
100,022,014	Bilirubin degradation product, C16H18N2O5 (3)**	Partially Characterized Molecules	Partially Characterized Molecules	0.059	−0.77
100,022,008	Bilirubin degradation product, C17H20N2O5 (1)**	Partially Characterized Molecules	Partially Characterized Molecules	0.062	−1.35
381	2-aminoadipate	Lysine Metabolism	Amino Acid	0.063	−0.60
1123	Chenodeoxycholate	Primary Bile Acid Metabolism	Lipid	0.070	1.09
100,001,755	4-vinylphenol sulfate	Benzoate Metabolism	Xenobiotics	0.072	1.08
100,002,749	S-methylcysteine	Methionine, Cysteine, SAM, and Taurine Metabolism	Amino Acid	0.074	−0.57
100,001,151	Butyrylglycine (C4)	Fatty Acid Metabolism (also BCAA Metabolism)	Lipid	0.074	−0.64
250	Biliverdin	Hemoglobin and Porphyrin Metabolism	Cofactors and Vitamins	0.074	−0.72
100,020,823	Lithocholate sulfate (1)	Secondary Bile Acid Metabolism	Lipid	0.075	−1.28
1526	1-palmitoyl-2-oleoyl-GPE (16:0/18:1)	Phosphatidylethanolamine (PE)	Lipid	0.075	−0.68
100,008,990	1-palmitoyl-2-arachidonoyl-GPE (16:0/20:4)*	Phosphatidylethanolamine (PE)	Lipid	0.076	−0.58
235	2-hydroxyphenylacetate	Phenylalanine Metabolism	Amino Acid	0.076	−0.67
100,001,617	Undecanedioate (C11-DC)	Fatty Acid, Dicarboxylate	Lipid	0.077	0.58
100,015,643	Sphingadienine	Sphingolipid Synthesis	Lipid	0.078	1.48
100,022,005	Bilirubin degradation product, C17H18N2O4 (1)**	Partially Characterized Molecules	Partially Characterized Molecules	0.081	−0.95
100,001,987	5alpha-androstan-3beta,17beta-diol disulfate	Androgenic Steroids	Lipid	0.083	−1.13
100,020,519	(2,4 or 2,5)-dimethylphenol sulfate	Food Component/Plant	Xenobiotics	0.087	0.81
100,010,919	Oleoyl-oleoyl-glycerol (18:1/18:1) [2]*	Diacylglycerol	Lipid	0.087	−0.84
100,022,009	Bilirubin degradation product, C17H20N2O5 (2)**	Partially Characterized Molecules	Partially Characterized Molecules	0.088	−1.17
100,022,015	Bilirubin degradation product, C16H18N2O5 (4)**	Partially Characterized Molecules	Partially Characterized Molecules	0.090	−0.91
100,001,413	N4-acetylcytidine	Pyrimidine Metabolism, Cytidine Containing	Nucleotide	0.090	−0.55
100,020,837	2,6-dihydroxybenzoic acid	Drug - Topical Agents	Xenobiotics	0.091	−0.89
100,001,950	Bilirubin (E, E)*	Hemoglobin and Porphyrin Metabolism	Cofactors and Vitamins	0.091	−1.10
100,002,137	5-hete	Eicosanoid	Lipid	0.091	1.56
100,010,918	Oleoyl-oleoyl-glycerol (18:1/18:1) [1]*	Diacylglycerol	Lipid	0.093	−0.98
100,004,110	3-methyl catechol sulfate (2)	Benzoate Metabolism	Xenobiotics	0.094	0.97
111	3-hydroxyisobutyrate	Leucine, Isoleucine, and Valine Metabolism	Amino Acid	0.094	−0.66
297	Sphingosine	Sphingosines	Lipid	0.094	1.43
100,008,984	1-palmitoyl-2-palmitoleoyl-GPC (16:0/16:1)*	Phosphatidylcholine (PC)	Lipid	0.095	−0.59
132	3-phosphoglycerate	Glycolysis, Gluconeogenesis, and Pyruvate Metabolism	Carbohydrate	0.098	0.84
999,907,765	X-07765			0.015	1.24
999,911,795	X-11,795			0.052	−0.70
999,912,112	X-12,112			0.007	−1.48
999,912,851	X-12,851			0.051	−1.63
999,914,939	X-14,939			0.066	0.53
999,915,245	X-15,245			0.026	−1.70
999,915,486	X-15,486			0.069	0.56
999,917,010	X-17,010			0.033	0.98
999,917,351	X-17,351			0.098	−1.01
999,918,901	X-18,901			0.024	−0.99
999,918,921	X-18,921			0.042	0.56
999,921,319	X-21,319			0.077	0.58
999,922,520	X-22,520			0.092	−0.81
999,924,295	X-24,295			0.061	−0.90
999,924,307	X-24,307			0.067	−0.61
999,924,344	X-24,344			0.023	1.00
999,924,425	X-24,425			0.026	1.21
999,924,456	X-24,456			0.025	0.68
999,924,565	X-24,565			0.018	1.61
999,925,519	X-25,519			0.078	1.16
999,925,520	X-25,520			0.028	−1.03
999,925,810	X-25,810			0.074	−0.76

*Log2FC:* log 2 fold change, *TCA:* tricarboxylic acid, *BCAA:* branched-chain amino acids

Elevated 3-sulfo-alanine (Log2FC = 1.47; *P* = 0.003) in ABN group suggesting perturbation in methionine and cysteine metabolism. N-acetyl-isoputreanine and N2-acetyl, N6,N6-dimethyllysine were downregulated in ABN group, indicating potential disruption in polyamine and lysine metabolic pathways. Sebacate (C10-DC) (Log2FC = 1.33; *P* = 0.005) and Suberate (C8-DC) (Log2FC = 0.87; *P* = 0.021) showed increased levels in ABN group, pointing to enhanced fatty acid dicarboxylate turnover. In contrast, several phospholipids such as 1-palmitoyl-2-linoleoyl-GPE (16:0/18:2) (Log2FC = −0.81) were reduced in ABN group, suggesting altered membrane lipid remodeling. Significant reductions were observed in retinol (Vitamin A) (Log2FC = −0.54) and Alpha-tocopherol (Vitamin E) (Log2FC = −1.90) in ABN group, which may reflect increased oxidative stress or impaired antioxidant defense. Pyruvate levels were decreased (Log2FC = −0.86; *P* = 0.016) in ABN group, indicating potential dysregulation in glycolysis or mitochondrial metabolism. Salicylate was downregulated (Log2FC = −1.35; *P* = 0.021) in ABN group, suggesting altered drug/xenobiotic processing.

#### Pre- and post-differences

The second analytical strategy evaluated metabolic changes between baseline and post-first chemotherapy cycle to refine biomarker discovery (Fig. 1). Using linear mixed models (LMM) adjusted for age, BMI, and race, we identified 78 metabolites with significant interaction effects between cardiac function groups (NL vs. ABN) and time (baseline vs. after the 1 st cycle of chemotherapy; Table 3; Fig. 4B). Of these, 67 were annotated and 11 unannotated; 9 metabolites showed negative effect sizes (upregulated in the ABN group), whereas 69 metabolites showed positive effect sizes (downregulated in the ABN group).

**Table 3:** Metabolite markers identified from Pre- and post- differences after first cycle of DOX-containing chemotherapy by linear mixed effects model (*P*-value < 0.05)

Chem ID	Name	Sub Pathway	Super Pathway	*P*-value	Effect
50	Spermidine	Polyamine Metabolism	Amino Acid	0.024	1.51
117	Homovanillate (HVA)	Tyrosine Metabolism	Amino Acid	0.038	−0.80
132	3-phosphoglycerate	Glycolysis, Gluconeogenesis, and Pyruvate Metabolism	Carbohydrate	0.020	0.94
209	AMP	Purine Metabolism, Adenine Containing	Nucleotide	0.014	1.42
235	2-hydroxyphenylacetate	Phenylalanine Metabolism	Amino Acid	0.038	−0.57
361	Inosine	Purine Metabolism, (Hypo)Xanthine/Inosine Containing	Nucleotide	0.006	1.67
363	Myo-inositol	Inositol Metabolism	Lipid	0.007	0.50
409	Malate	TCA Cycle	Energy	0.022	0.46
432	Nicotinamide	Nicotinate and Nicotinamide Metabolism	Cofactors and Vitamins	0.040	1.33
439	Stearate (18:0)	Long-Chain Saturated Fatty Acid	Lipid	0.041	0.26
445	Orotate	Pyrimidine Metabolism, Orotate containing	Nucleotide	0.001	0.63
512	Taurine	Methionine, Cysteine, SAM, and Taurine Metabolism	Amino Acid	0.024	0.68
798	Adenosine	Purine Metabolism, Adenine Containing	Nucleotide	0.016	0.65
1004	Xanthine	Purine Metabolism, (Hypo)Xanthine/Inosine Containing	Nucleotide	0.019	0.73
1022	Picolinate	Tryptophan Metabolism	Amino Acid	0.015	−0.97
1137	Oleoyl ethanolamide	Endocannabinoid	Lipid	0.033	0.48
1215	N-acetylglucosaminyl asparagine	Amino Sugar Metabolism	Carbohydrate	0.044	0.56
1231	Dihomolinoleate (20:2n6)	Long-Chain Polyunsaturated Fatty Acid (n3 and n6)	Lipid	0.030	0.51
1242	1-methyladenosine	Purine Metabolism, Adenine Containing	Nucleotide	0.048	0.15
1254	Glycerol	Glycerolipid Metabolism	Lipid	0.023	0.63
1488	Arachidonoyl ethanolamide	Endocannabinoid	Lipid	0.004	0.61
1489	Palmitoyl ethanolamide	Endocannabinoid	Lipid	0.007	0.43
100,000,016	Suberate (C8-DC)	Fatty Acid, Dicarboxylate	Lipid	0.015	0.95
100,000,639	1-stearoyl-2-oleoyl-GPS (18:0/18:1) (PE(18:0/18:1(9Z))	Phosphatidylserine (PS)	Lipid	0.029	2.02
100,000,776	Palmitoylcarnitine (C16)	Fatty Acid Metabolism (Acyl Carnitine, Long-Chain Saturated)	Lipid	0.043	0.26
100,000,802	Acetylcarnitine (C2)	Fatty Acid Metabolism (Acyl Carnitine, Short-Chain)	Lipid	0.007	0.33
100,001,102	Dodecanedioate (C12)	Fatty Acid, Dicarboxylate	Lipid	0.031	0.74
100,001,121	Pyridoxate	Vitamin B6 Metabolism	Cofactors and Vitamins	0.029	−0.54
100,001,145	3-hydroxysebacate	Fatty Acid, Monohydroxy	Lipid	0.031	0.72
100,001,195	Docosatrienoate (22:3n3)	Long-Chain Polyunsaturated Fatty Acid (n3 and n6)	Lipid	0.045	0.68
100,001,211	Sebacate (C10-DC)	Fatty Acid, Dicarboxylate	Lipid	0.006	1.00
100,001,416	Orotidine	Pyrimidine Metabolism, Orotate Containing	Nucleotide	0.030	0.34
100,001,454	3-Hydroxydodecanedioate*	Fatty Acid, Dicarboxylate	Lipid	0.019	0.86
100,001,612	N-acetyl-aspartyl-glutamate (NAAG)	Glutamate Metabolism	Amino Acid	0.024	0.46
100,001,614	Hexadecanedioate (C16)	Fatty Acid, Dicarboxylate	Lipid	0.006	0.63
100,001,740	Mannitol/sorbitol	Fructose, Mannose, and Galactose Metabolism	Carbohydrate	0.038	−0.99
100,001,810	Dimethylarginine (ADMA + SDMA)	Urea cycle; Arginine and Proline Metabolism	Amino Acid	0.030	0.36
100,002,196	13-HODE + 9-HODE	Fatty Acid, Monohydroxy	Lipid	0.029	0.96
100,002,199	Tridecenedioate (C13:1-DC)*	Fatty Acid, Dicarboxylate	Lipid	0.015	0.62
100,002,356	(16 or 17)-methylstearate (a19:0 or i19:0)	Fatty Acid, Branched	Lipid	0.042	0.40
100,002,953	16-hydroxypalmitate	Fatty Acid, Monohydroxy	Lipid	0.008	0.44
100,003,119	N-oleoyl taurine	Endocannabinoid	Lipid	0.016	0.60
100,003,271	Beta-citrylglutamate	Glutamate Metabolism	Amino Acid	0.031	0.79
100,003,686	N-palmitoyl glycine	Fatty Acid Metabolism (Acyl Glycine)	Lipid	0.038	0.32
100,003,926	(R)−3-hydroxybutyrylcarnitine	Fatty Acid Metabolism (Acyl Carnitine, Hydroxy)	Lipid	0.004	1.09
100,004,054	Margaroylcarnitine (C17)*	Fatty Acid Metabolism (Acyl Carnitine, Long-Chain Saturated)	Lipid	0.041	0.33
100,004,635	Methionine sulfone	Methionine, Cysteine, SAM, and Taurine Metabolism	Amino Acid	0.001	0.37
100,005,996	Octadecenedioylcarnitine (C18:1-DC)*	Fatty Acid Metabolism (Acyl Carnitine, Dicarboxylate)	Lipid	0.044	0.52
100,006,360	Dopamine 4-sulfate	Tyrosine Metabolism	Amino Acid	0.008	−1.24
100,006,361	Dopamine 3-O-sulfate	Tyrosine Metabolism	Amino Acid	0.017	−0.95
100,006,367	3-hydroxyhexanoate	Fatty Acid, Monohydroxy	Lipid	0.046	0.30
100,006,369	N-carbamoyl alanine	Alanine and Aspartate Metabolism	Amino Acid	0.042	−0.66
100,006,435	N-acetylglucosamine/N-acetyl galactosamine	Amino Sugar Metabolism	Carbohydrate	0.020	0.42
100,006,614	Adipoylcarnitine (C6-DC)	Fatty Acid Metabolism (Acyl Carnitine, Dicarboxylate)	Lipid	0.045	0.47
100,006,627	Suberoylcarnitine (C8-DC)	Fatty Acid Metabolism (Acyl Carnitine, Dicarboxylate)	Lipid	0.009	0.81
100,009,271	(S)−3-hydroxybutyrylcarnitine	Fatty Acid Metabolism (Acyl Carnitine, Hydroxy)	Lipid	0.016	0.68
100,010,949	Stearoyl-arachidonoyl-glycerol (18:0/20:4) [1]*	Diacylglycerol	Lipid	0.022	0.98
100,015,643	Sphingadienine	Sphingolipid Synthesis	Lipid	0.032	1.55
100,019,801	3-hydroxyoleoylcarnitine	Fatty Acid Metabolism (Acyl Carnitine, Hydroxy)	Lipid	0.038	0.59
100,019,966	3-hydroxyoleate*	Fatty Acid, Monohydroxy	Lipid	0.049	0.63
100,019,972	Dodecenedioate (C12:1-DC)*	Fatty Acid, Dicarboxylate	Lipid	0.026	0.82
100,019,975	Hexadecenedioate (C16:1-DC)*	Fatty Acid, Dicarboxylate	Lipid	0.042	0.55
100,019,978	Octadecenedioate (C18:1-DC)	Fatty Acid, Dicarboxylate	Lipid	0.038	0.51
100,021,100	Tetradecadienedioate(C14:2-DC)*	Fatty Acid, Dicarboxylate	Lipid	0.011	0.94
100,021,709	Branched-chain, straight-chain, or cyclopropyl 12:1 fatty acid*	Partially Characterized Molecules	Partially Characterized Molecules	0.029	0.62
100,021,711	Decadienedioic acid (C10:2-DC)**	Fatty Acid, Dicarboxylate	Lipid	0.018	0.98
100,022,120	Oxindolylalanine	Tryptophan Metabolism	Amino Acid	0.016	0.49
999,911,299	X-11,299			0.026	0.77
999,911,483	X-11,483			0.039	0.63
999,912,729	X-12,729			0.007	−1.48
999,913,007	X-13,007			0.024	0.95
999,914,904	X-14,904			0.027	0.98
999,923,641	X-23,641			0.042	0.57
999,923,665	X-23,665			0.046	0.66
999,924,456	X-24,456			0.032	0.46
999,924,565	X-24,565			0.006	1.90
999,926,107	X-26,107			0.019	0.37
999,926,111	X-26,111			0.041	0.48

*AMP:* adenosine monophosphate, *TCA:* tricarboxylic acid

Venn diagram analysis highlighted ten metabolites common to both baseline (T0) and pre-post comparisons (T0-T1; Fig. 4C), including 3-phosphoglycerate, 2-hydroxyphenylacetate, inosine, taurine, suberate (C8-DC), sebacate (C10-DC), sphingadienine, oxindolylalanine, x-24456, and x-24565. From the Venn diagram, 90 metabolites were unique to baseline (e.g., 1-palmitoyl-2-linoleoyl-GPE (16:0/18:2), 5-methylthioadenosine (MTA), dihomo-linoleoylcarnitine (C20:2), dihomo-linolenoylcarnitine (C20:3n3 or 6), and 1-oleoyl-2-linoleoyl-GPE (18:1/18:2), while 68 metabolites were exclusive to pre-post differences (e.g., myo-inositol, dimethylarginine (ADMA + SDMA), N-acetylglucosaminyl asparagine, N-acetyl-aspartyl-glutamate (NAAG), and N-acetyl glucosamine/N-acetyl galactosamine). These findings suggest chemotherapy-induced metabolic shifts that may contribute to increased cardiotoxicity risk in breast cancer patients.

### Machine learning–based validation of metabolomic findings

**Fig. 5:**
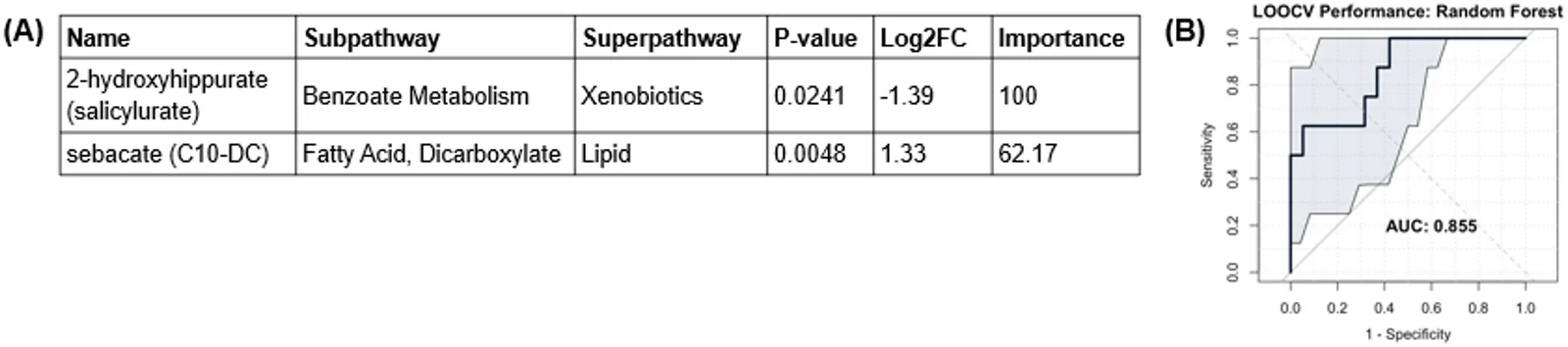
Multi-marker analysis with RandomForest modeling at baseline. **(A)** Metabolites with |Log2FC| ≥ 0.5 and *P*-value < 0.1 in ANOVA test of comparison at baseline were used as input. Two metabolites -2-hydroxyhippurate (salicylurate) and sebacate (C10-DC) were identified in the final Random Forest model with importance score of 100 and 62.17, respectively. **(B)** ROC curve from LOOCV-based Random Forest analysis showing moderate discrimination (AUC = 0.855)

Random Forest modeling was applied to evaluate the discriminatory performance of metabolite markers identified at baseline and from pre–post chemotherapy comparisons. Feature sets from each time-points were analyzed independently, with candidate metabolites first reduced using stepwise logistic regression. At baseline, metabolites selected from ANOVA-based comparisons (|Log2FC| ≥ 0.5 and *P* < 0.1) were used as input features. Two metabolites-sebacate (C10-DC) (Log2FC = 1.33; *P* = 0.005) and 2-hydroxyhippurate (salicylurate) (Log2FC = − 1.39; *P* = 0.024) were identified as the top candidate predictors of cardiotoxicity, with relative importance scores of 100 and 62.17, respectively (Fig. 5A). Using leave-one-out cross-validation (LOOCV), the RF model achieved optimal performance with an mtry value of 2, yielding an accuracy of 81.5%, which is notable given the small cohort (*n* = 27). Receiver operating characteristic (ROC) analysis demonstrated moderate discriminatory performance, with an AUC of 0.855. These models were used to explore discriminatory potential rather than to establish clinically applicable predictive models.

The model exhibited high specificity (89.5%) in identifying normal (NL) cases but moderate sensitivity (62.5%) for detecting abnormal (ABN) cases (Fig. 5B). The relatively lower sensitivity indicates limited ability to detect ABN cases, likely due to small sample size and class imbalance. The P-value comparing model accuracy to the No Information Rate (0.1449) indicates that performance did not reach statistical significance at the 0.05 level, likely due to limited sample size.

Additionally, Random Forest analyses were conducted to evaluate candidate metabolites associated with left ventricular ejection fraction (LVEF) decline and to validate markers identified from pre–post chemotherapy changes. These results should be considered exploratory due to the small sample size.

**Fig. 6:**
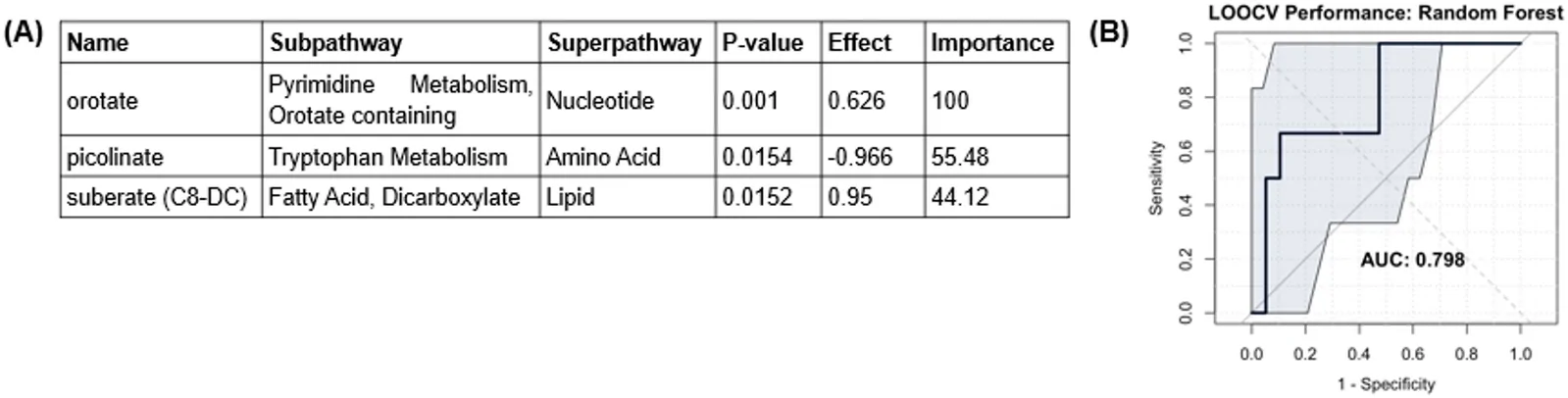
Multi-marker Random Forest Analysis of pre-post difference after 1 st DOX-containing chemotherapy (T0-T1). **(A)** Metabolites with *P*-value < 0.05 from an interaction of cardiotoxicity and time variables in the linear mixed model of pre-post design were used as an input. Three metabolites-orotate, picolinate, and suberate (C8-DC) were identified in the final Random Forest model with importance 100, 55.48, and 44.12. **(B)** ROC curve from LOOCV-based Random Forest analysis showing moderate discrimination (AUC = 0.798)

Metabolites selected from the second approach based on pre- and post- differences were used as inputs in the model. Three metabolites: orotate (effect size = 0.626; *P* =0.001), picolinate (effect size = − 0.966; *P* = 0.015), and suberate (C8-DC) (effect size = 0.95; *P* = 0.015) were identified as key candidate predictors, with relative importance scores of 100, 55.48, and 44.12, respectively (Fig. 6A). Using LOOCV, the Random Forest model achieved optimal performance with an *mtry* value of 2, yielding an accuracy of 80%. ROC analysis showed moderate performance (AUC = 0.798 (Fig. 6B). Increasing the *mtry* to 3 or 4 parameters resulted in lower accuracies (76%), indicating that a more constrained model performed better in this small cohort. While specificity remained high (89.5%), sensitivity for detecting ABN cases was low (50%). The P-value for accuracy versus the No Information Rate (0.42) further indicates limited statistical significance. Overall, the model lacks sufficient sensitivity for reliable detection of abnormal cases in this small cohort.

Across both analyses, metabolites such as sebacate (C10-DC), 2-hydroxyhippurate, orotate, picolinate, and suberate (C8-DC) consistently emerged as candidate predictors of DIC. These findings highlight the potential of metabolomic profiling, combined with machine learning approaches, to identify candidate biomarkers of cardiotoxicity. However, given the limited sample size, these results should be interpreted as exploratory and require validation in larger, independent cohorts. Collectively, these metabolites suggest involvement of pathways related to nucleotide metabolism, fatty acid oxidation, and xenobiotic processing, underscoring their potential clinical relevance for early detection and monitoring of cardiotoxicity.

### Pathway enrichment analysis

**Fig. 7:**
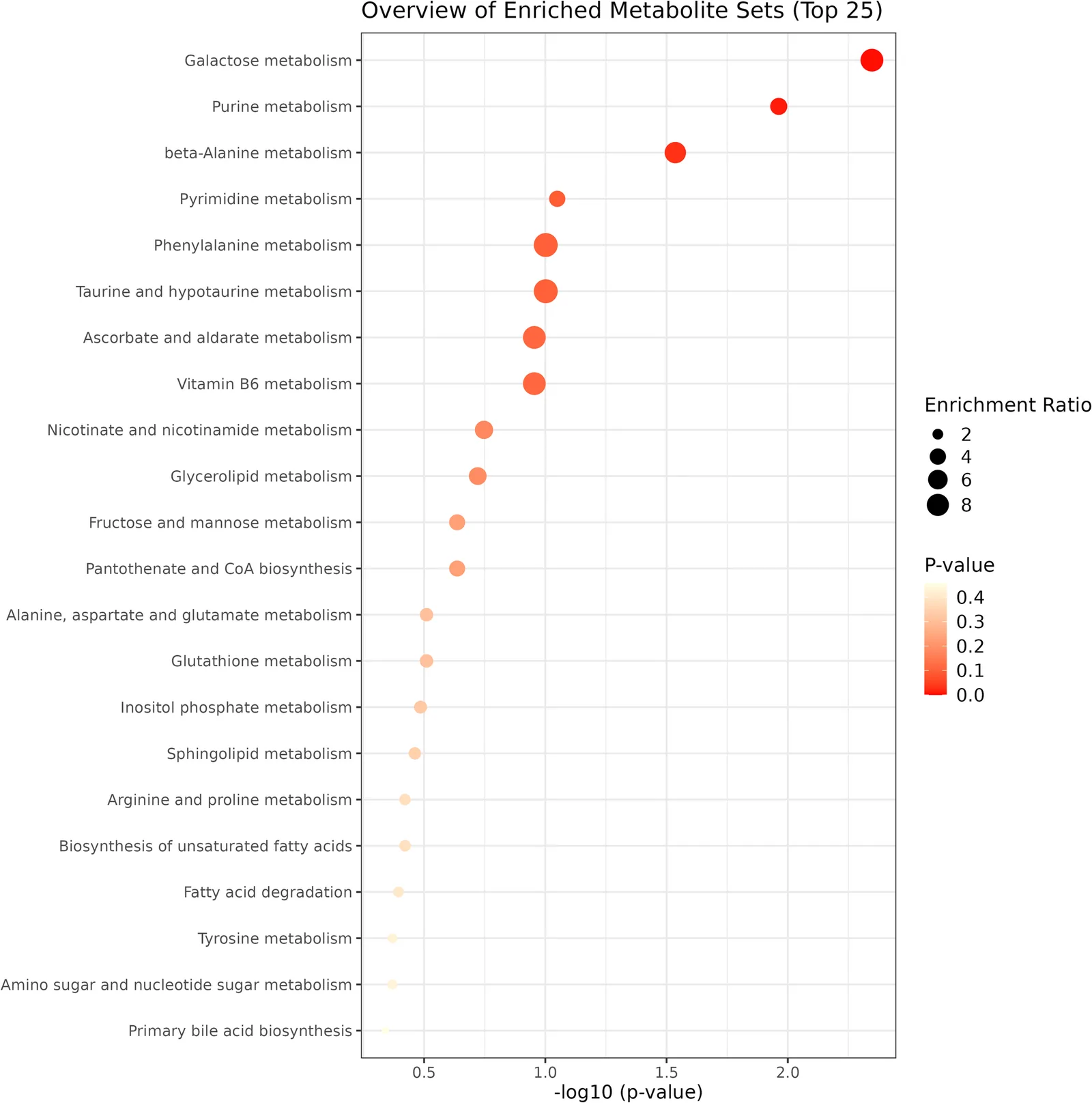
Pathway enrichment analysis of metabolic perturbations linked to DOX-induced cardiotoxicity in patients with breast cancer

To investigate the metabolic pathways potentially involved in DIC among breast cancer patients, we conducted pathway enrichment analysis using 78 metabolites identified from pre-post chemotherapy comparisons. Of these, 59 metabolites were successfully mapped to Human Metabolome Database (HMDB) IDs (https://hmdb.ca/) and included in the MetPA analysis (www.metaboanalyst.ca). Pathway enrichment analysis revealed three significantly perturbed pathways associated with DIC: galactose metabolism, involving metabolites such as glycerol, sorbitol, and myo-inositol; purine metabolism, characterized by alterations in xanthine, adenosine monophosphate, adenosine, and inosine; and beta-alanine metabolism, with significant changes observed in ureidopropionic acid and spermidine **(**Fig. 7**).** Detailed pathway enrichment results are displayed in Supplemental Table S1. Collectively, these findings suggest that disruptions in carbohydrate, nucleotide, and amino acid metabolism may play critical roles in the metabolic adaptations or disruptions associated with DIC.

## Discussion

Cardiotoxicity remains a critical challenge in the treatment of breast cancer, often manifesting as subclinical cardiac dysfunction that escapes early detection by conventional imaging. In this study, we applied plasma metabolomics to blood samples collected from breast cancer patients undergoing DOX-containing chemotherapy, stratified by normal and abnormal LVEF. We then used plasma metabolomics to investigate metabolic alterations linked to DIC. Despite similar demographic characteristics at baseline, including age and BMI, the two groups displayed distinct metabolic signatures, which became more pronounced following statistical and machine learning analyses, highlighting the sensitivity of metabolomics in detecting subtle biochemical changes associated with early cardiac injury that conventional clinical measures often fail to detect.

By comparing baseline and post-first cycle of chemotherapy (pre-post) metabolomic profiles, we identified metabolites and pathways linked to DIC. Metabolic separation between groups was evident both at baseline and in pre-post comparisons, underscoring the dynamic nature of chemotherapy-induced metabolic shifts. Among 100 significant metabolites at baseline and 78 at pre-post comparisons, 10 metabolites were overlapped, providing a focused list of candidate biomarkers. Machine learning models identified sebacate and 2-hydroxyhippurate as key candidate predictors at baseline, whereas orotate, picolinate, and suberate emerged as significant markers of pre-post changes following the first chemotherapy cycle. These metabolites demonstrated moderate predictive performance, with relatively high specificity but lower sensitivity, reflecting limited ability to detect abnormal (ABN) cases in this small cohort. This imbalance indicates that the models are more effective at identifying normal cases than detecting cardiotoxicity, suggesting potential utility as candidate indicators of cardiotoxicity that require validation in larger cohorts. However, these findings should be interpreted cautiously given the limited sample size and exploratory nature of the analysis.

A significant shift in lipid metabolism was observed in the study. Of the 78 metabolites identified after the first cycle of chemotherapy, 39 were related to lipid metabolism. These metabolites are involved in acylcarnitine metabolism, fatty acid dicarboxylates, monohydroxy fatty acids, glycerophospholipids, inositol metabolism, long-chain fatty acids, phospholipids, sphingolipids, endocannabinoids, and diacylglycerols. Acylcarnitines are integral to cellular metabolism and energy production, facilitating the transport of long-chain fatty acids into mitochondria for β-oxidation, a process critical for balancing intracellular sugar and lipid metabolism (Makrecka et al., 2014; McCoin et al., 2015). Disruptions in this process, such as impaired fatty acid oxidation or mitochondrial dysfunction, can lead to acylcarnitine accumulation, resulting in lipotoxicity, oxidative stress, and reduced ATP production (Calvani et al., 2000; Dambrova et al., 2022; McCoin et al., 2015). The heart, which relies heavily on fatty acid oxidation, is particularly sensitive to these disturbances, increasing vulnerability to cardiotoxicity under such conditions (Makrecka et al., 2014; McCoin et al., 2015). Altered acylcarnitine profiles have been linked to risk of cardiovascular disease among diabetic patients (Zhao et al., 2021), patients with non-obstructive coronary artery disease, both revealed that elevated levels are linked to higher long-term risk of cardiovascular disease and all-cause mortality (Storesund et al., 2022). In patients with heart failure, elevated acylcarnitines, such as hydroxybutyrylcarnitine and octadecenoylcarnitine, have shown prognostic value beyond traditional biomarkers (Cheng et al., 2015). In this study, decreased levels of both long-chain and short-chain acylcarnitines (e.g., adipoylcarnitine [C6-DC], octadecenedioylcarnitine [C18:1-DC], suberoylcarnitine [C8-DC], palmitoylcarnitine [C16:0], and hydroxybutyrylcarnitine) were observed after post-first cycle of chemotherapy (pre-post) among ABN group, These findings are consistent with previous work in doxorubicin-treated HER2-positive and HER2-negative breast cancer patients (Thonusin et al., 2024). These results reinforce the link between acylcarnitine and cardiovascular pathology, particularly in the context of breast cancer therapies that may disrupt mitochondrial function or fatty acid metabolism.

Dicarboxylic acids (DCAs) play a complex role in cardiac health and metabolism, undergo β-oxidation in both peroxisomes and mitochondria through a carnitine-independent pathway, particularly in conditions like metabolic stress, helping to mitigate toxic fatty acid accumulation (Ranea-Robles & Houten, 2023; Ruiz-Sala & Peña-Quintana, 2021). Medium-chain, water-soluble DCAs are used in parenteral nutrition (Fürstenberger et al., 2006) and provide energy to cardiac and skeletal muscles (Bharathi et al., 2020; Pourfarzam & Bartlett, 1993). Among these, dodecanedioic acid is notable for its high energy density and minimal urinary loss (Grego & Mingrone, 1995). However, excessive accumulation of DCAs may indicate fatty acid overflow, triggering oxidative stress and contributing to cardiac dysfunction over time. When mitochondrial β-oxidation is impaired, fatty acids can be metabolized through an alternate pathway called omega-oxidation, which occurs in the endoplasmic reticulum. This compensatory mechanism results in the production of DCAs such as suberic acid and may reflect insufficient carnitine availability or a deficiency in nutrient cofactors necessary for efficient β-oxidation (Ranea-Robles & Houten, 2023). In this study, elevated levels of DCAs, including sebacate and suberate, were already present at baseline, suggesting a pre-existing impairment in β-oxidation prior to the initiation of chemotherapy. Elevated levels of DCAs, which are linked to increased peroxisomal oxidation, can exacerbate mitochondrial strain, impair contractility, and promote cardiac dysfunction and cell death (Zhang et al., 2023). Similar DCA elevations have been reported in conditions such as chronic thromboembolic pulmonary hypertension and heart failure with depression (Heresi et al., 2020), and higher levels of sebacate and suberate have been observed in patients with heart failure and depression compared to those without (Steffens et al., 2010). Collectively, these findings highlight the complex metabolic roles of DCAs in cardiovascular disease.

Among the most significant findings, picolinate, a metabolite in the tryptophan-kynurenine pathway, showed increased levels in the ABN group after the first cycle of chemotherapy. Elevated picolinate levels have previously been observed in coronary heart disease (Santisukwongchote et al., 2020), and are part of a broader class of metabolites known as kynurenines, which also includes 3-hydroxykynurenine, xanthurenic acid, anthranilic acid, and quinolinic acid collectively referred to as kynurenines. The kynurenine pathway is known to generate both neuroprotective and neurotoxic compounds (Grant et al., 2009), and its dysregulation has been increasingly implicated in neurodegenerative diseases, cognitive impairment, and chemotherapy-related neurotoxicity (Grant et al., 2009; Maddison & Giorgini, 2015; Sordillo & Sordillo, 2020; Tan et al., 2012; Wang et al., 2025). Anthracycline-based regimens (e.g., doxorubicin) are associated with cardiotoxicity rates ranging from 4% to 18%, depending on cumulative dose and patient risk factors (Papageorgiou et al., 2021). In parallel, neurotoxicity, particularly chemotherapy-related cognitive impairment, affects 17–75% of patients, with a subset experiencing long-term deficits (Kamińska & Cudnoch-Jędrzejewska, 2023). Although doxorubicin has limited direct penetration of the blood–brain barrier, it can induce systemic inflammation, oxidative stress, and mitochondrial dysfunction, which may contribute to central nervous system effects. These findings suggest that systemic metabolic alterations associated with the kynurenine pathway may contribute to DOX-induced toxicity; however, the specific tissue origin of these changes remains unclear and requires further investigation.

Orotic acid (orotate), a crucial intermediate in pyrimidine biosynthesis, has been implicated in endothelial dysfunction and hypertension when dysregulated. Elevated plasma orotate levels result from inborn errors in pyrimidine biosynthesis, particular mutations in uridine 5′-monophosphate synthase, the enzyme responsible for converting orotate into uridine 5′-monophosphate (Smith et al., 1961). High orotate concentrations have been shown to impair endothelial function by disrupting insulin- and metformin-induced nitric oxide production and endothelial nitric oxide synthase (eNOS) phosphorylation in endothelial cells. This impairment is linked to reduced PI3K-Akt signaling, a pathway crucial for stimulating eNOS-mediated nitric oxide production. Additionally, activation of AMP-activated protein kinase enhances eNOS phosphorylation, underscoring the critical role of AMP-activated protein kinase in vascular homeostasis (Choi et al., 2015). Our findings align with prior in vivo studies demonstrating the clinical relevance of orotate dysregulation (Choi et al., 2015). Notably, our study found higher baseline levels of orotate in ABN patients, suggesting the presence of subclinical endothelial impairment even before chemotherapy initiation. These insights suggest that orotate dysregulation may contribute to cardiovascular complications, by exacerbating endothelial dysfunction and impairing vascular signaling.

Other significant findings from our plasma metabolomic analysis suggest systemic metabolic alterations associated with DOX treatment and LVEF decline. Elevated levels of taurine, known for its cardioprotective and anti-inflammatory effects, in this study reduced taurine level in ABN group, after first cycle of chemotherapy, may reflect a non-compensatory response to DOX-induced stress and osmotic imbalance (Moloney et al., 2010; Shoaib et al., 2020; Tzang et al., 2024). Elevated baseline levels of dimethylarginines (ADMA and SDMA), endogenous inhibitors of nitric oxide synthase, are linked to endothelial dysfunction and reduced nitric oxide bioavailability, contributing to impaired cardiac perfusion (Hsu et al., 2016; Liu et al., 2016) (Winkler et al., 2018), were reduced after the first chemotherapy cycle, suggesting a potential normalization of nitric oxide–mediated vascular function during early treatment. Disruptions in glycosylation pathways, including elevated baseline level of N-acetylglucosamine/N-acetylgalactosamine and N-acetylglucosaminylasparagine, suggest altered protein modification and stress signaling in cardiomyocytes (Gravina et al., 2022; Ha et al., 2023; Jones et al., 2008), Their reduction after the first chemotherapy cycle may reflect an adaptive metabolic response aimed at mitigating glycosylation stress and preserving cardiomyocyte function. The presence of NAAG, a glutamate source in high-grade cancers, may indicate metabolic crosstalk between cancer progression and cardiac function, although its role in cardiotoxicity remains unclear (Nguyen et al., 2019). Elevated baseline levels of 3-phosphoglycerate, a key intermediate in glycolysis and serine biosynthesis, points to a metabolic shift toward glycolysis and impaired mitochondrial ATP production (Badolia et al. 2020; Chen et al. 2023b; Oslund et al. 2017; Tao et al. 2024), were reduced after first cycle of chemotherapy. Altered levels of dopamine sulfate conjugates may reflect changes in sympathetic activity and stress adaptation mechanisms (Mahbubul Huq et al., 1988). Increased myo-inositol at baseline, involved in membrane signaling and gene expression, has been associated with heart failure and may contribute to hypertrophy and fibrosis (Berridge, 2009, 2016; Pouleur et al., 2024), was reduced after the first cycle of chemotherapy. This shift highlights myo-inositol as a potential biomarker of early cardiac response to chemotherapy and warrants further investigation into its prognostic value. Elevated bioactive lipids such as OEA and PEA at baseline, which exhibit anti-inflammatory and metabolic benefits, further support their potential role in mitigating cardiac damage (Comella et al., 2024; Rinne et al., 2018; Sabahi et al., 2022; Wu et al., 2017), was reduced after the first cycle of chemotherapy. This decline may indicate a loss of endogenous cardioprotective capacity during treatment, potentially increasing vulnerability to chemotherapy-induced cardiac injury.

**Fig. 8:**
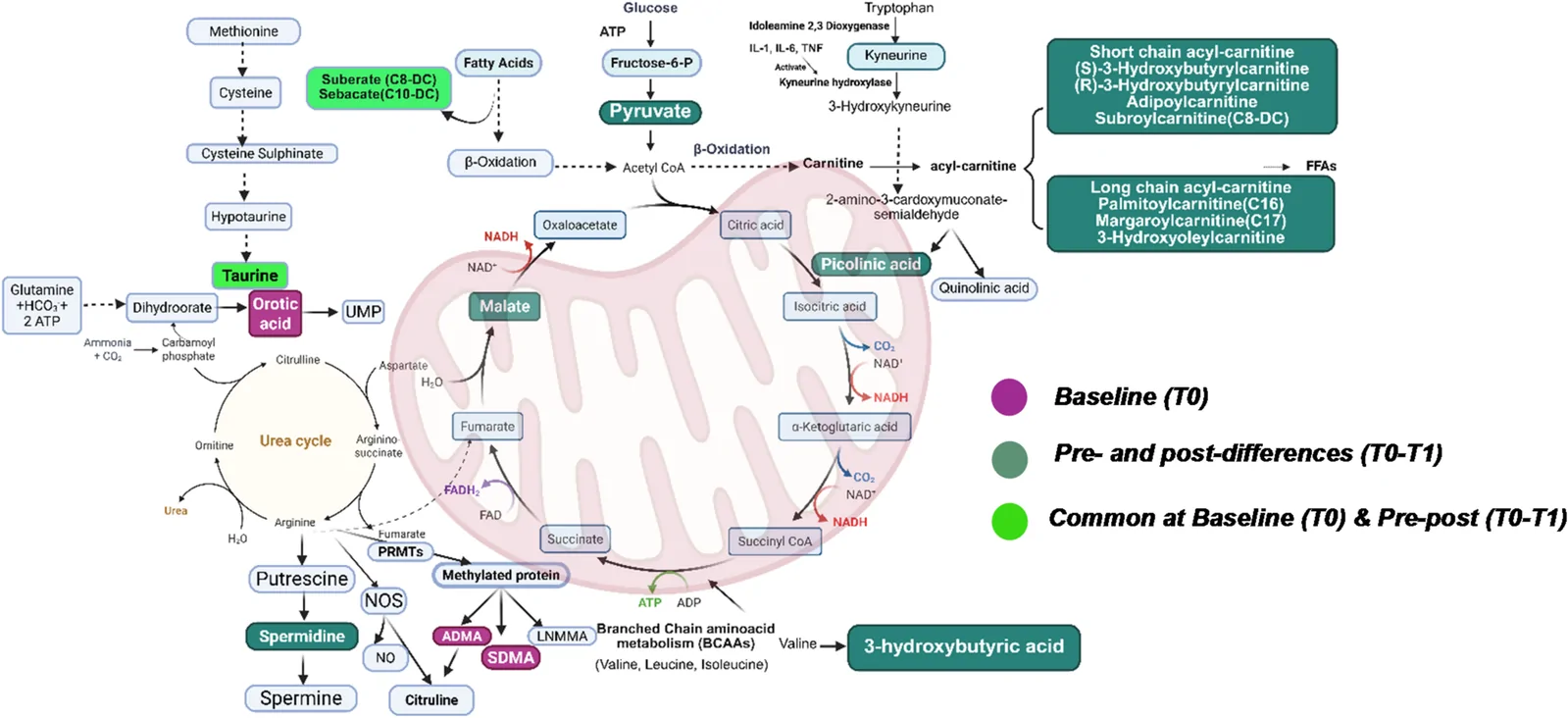
Schematic representation of circulating metabolite perturbations associated with doxorubicin treatment and LVEF decline in patients with breast cancer

Our study revealed significant alterations in multiple metabolic pathways following treatment with DOX, highlighting its impact on metabolic processes (Fig. 8). Importantly, these pathway-level disruptions are supported by changes in key metabolites identified in our analysis. For example, sebacate and suberate, which emerged as candidate predictors, are dicarboxylic acids involved in fatty acid ω-oxidation and peroxisomal metabolism, linking them to broader alterations in lipid metabolic pathways. Similarly, picolinate, a metabolite within the tryptophan–kynurenine pathway, reflects perturbations in amino acid metabolism and stress-response signaling (Badawy, 2017; Wang et al., 2025), while orotate, a key intermediate in pyrimidine biosynthesis, aligns with disruptions in nucleotide metabolism. Pathway enrichment analysis identified significant disruptions in galactose metabolism, purine metabolism, and beta-alanine metabolism. Alterations in galactose metabolism, particularly involving sorbitol and myo-inositol, suggest mechanisms of cardiac stress. Sorbitol pathway activity, implicated in tissue complications, may play a role in DIC, necessitating further investigation. Disruptions in purine metabolism, characterized by changes in metabolites like xanthine and adenosine emphasize potential impacts on cardiac energy metabolism and oxidative stress, which may exacerbate ischemia-reperfusion injury, a common complication of DOX treatment. Additionally, perturbations in beta-alanine metabolism, including cardioprotective compounds like spermidine, indicate a potential compensatory response to DOX-induced stress. These findings provide insights into DOX-induced metabolic changes and offer potential avenues for mitigating cardiotoxicity. These findings are consistent with recent metabolomic research that emphasizes the utility of pathway-level analysis and biomarker identification in understanding anthracycline-induced cardiotoxicity (Singh et al., 2025). Targeting affected pathways or supplementing protective metabolites may inform future studies aimed at reducing cardiac complications, while preserving the anticancer efficacy of DOX. The study also underscores the value of metabolomic profiling for early detection and risk stratification of cardiotoxicity, paving the way for personalized treatment strategies in breast cancer patients undergoing chemotherapy. These pathway-level findings provide biological context for the identified metabolites, suggesting coordinated metabolic disruption rather than isolated biomarker effects.

Despite these promising findings, several limitations should be considered. The relatively small sample size limits statistical power and increases the risk of overfitting. Concomitant medication use represents a potential source of confounding, as patients with hypertension continued antihypertensive therapy (e.g., β-blockers, ACE inhibitors), and patients with diabetes continued treatment with insulin or metformin during chemotherapy. These medications may influence systemic metabolomic profiles, particularly pathways related to glucose metabolism, lipid metabolism, and mitochondrial function. In our cohort, hypertension was similarly distributed between groups, whereas diabetes was present only in the NL group; however, these differences were not statistically significant. Given the limited sample size, we were unable to adjust for medication-specific effects, and residual confounding cannot be excluded.

In addition, because plasma metabolomics reflects systemic metabolic activity, the observed metabolite changes should be interpreted as circulating biomarkers associated with LVEF decline rather than direct evidence of cardiomyocyte-specific injury. The high-dimensional nature of metabolomics data, combined with a relatively small sample size, increases the risk of multiple-testing bias, model overfitting, and false-positive findings. The use of relatively lenient screening thresholds (e.g., *P* < 0.1 in the baseline analysis) may further increase the likelihood of identifying chance associations. Although Benjamini–Hochberg false discovery rate (FDR) correction was considered, it was not applied due to the exploratory nature of this study and limited statistical power; consequently, the reported metabolite associations should be interpreted as preliminary and hypothesis-generating rather than definitive.

To mitigate overfitting, we implemented a leave-one-out cross-validation (LOOCV) framework for Random Forest modeling, allowing each sample to contribute to both training and validation. While LOOCV maximizes data utilization in small cohorts, it may still produce optimistic performance estimates and does not substitute for independent external validation. Additionally, the limited number of ABN cases may contribute to imbalanced model performance, as reflected by high specificity but lower sensitivity, indicating that the model is more effective at identifying NL cases than detecting ABN cases. Although we applied a multi-step analytical workflow incorporating complementary statistical approaches, feature selection, and machine learning-based validation, recent studies have demonstrated that integrating machine learning models with advanced interpretability approaches can enhance predictive performance and clinical applicability in breast cancer research (Yuan et al., 2025), However, these approaches do not fully eliminate the risk of overfitting in small cohorts. Recent pharmacovigilance research has emphasized the importance of systematic monitoring of adverse drug reactions associated with breast cancer therapies, including neurologic adverse events such as seizures linked to novel antineoplastic agents (Sha et al., 2025), these findings emphasize the importance of systematic monitoring and early detection strategies for therapy-related toxicity. Taken together, our results support the potential utility of metabolomic profiling as a tool for early detection and risk stratification of chemotherapy-induced cardiotoxicity.

## Conclusion

Cardiotoxicity remains a significant challenge in chemotherapy, with agents like DOX inducing cardiac damage through diverse mechanisms and variable clinical presentations. In this study, we employed plasma metabolomics to investigate the impact of DOX treatment on the metabolomic profiles of patients with breast cancer before and after chemotherapy. Our findings identified key metabolites, including sebacate, 2-hydroxyhippurate, orotate, picolinate, and suberate, as well as disruptions in galactose, purine, and beta-alanine metabolic pathways associated with DIC. These findings provide preliminary insights that may inform future studies aimed at developing targeted strategies for early detection and intervention for DIC, potentially enhancing long-term cardiac outcomes. Integrating metabolomic biomarkers into clinical practice could facilitate a personalized approach to chemotherapy management, minimizing cardiotoxic risks while preserving oncological efficacy. This aligns with recent work emphasizing the role of metabolomics in identifying predictive biomarkers and mechanistic insights into anthracycline-induced cardiotoxicity. Overall, our findings underscore the potential utility of metabolomic profiling in managing chemotherapy-induced cardiotoxicity in breast cancer patients.

## Supplementary Information

Below is the link to the electronic supplementary material.

Supplementary Material 1

Supplementary Material 2

## Data Availability

No datasets were generated or analysed during the current study.
