# Supplementary material for: Metabolic phenotypes of doxorubicin-induced cardiotoxicity among patients with breast cancer
Source: Metabolomics. 2026 Jul 4;22(4):119. doi: 10.1007/s11306-026-02469-7 (PMC13332971; doi:10.1007/s11306-026-02469-7)
Supplement: Supplementary file 1 — Supplementary Material 1 [file 11306_2026_2469_MOESM1_ESM.docx]

**Supplemental Table S1.** Enrichment analysis of metabolomic pathways perturbed by doxorubicin-induced cardiotoxicity

| **Pathways** | **Total** | **Expected** | **Hits** | **Raw P** | **FDR** |
| --- | --- | --- | --- | --- | --- |
| Galactose metabolism | 27 | 0.351 | Glycerol, Sorbitol; myo-Inositol | 0.0045 | 0.36 |
| Purine metabolism | 70 | 0.91 | Xanthine, Adenosine monophosphate, Adenosine, Inosine | 0.0109 | 0.438 |
| β-Alanine metabolism | 21 | 0.273 | Ureidopropionic acid, Spermidine | 0.0291 | 0.775 |
| Pyrimidine metabolism | 39 | 0.507 | Ureidopropionic acid, Orotic acid | 0.0893 | 1 |
| Phenylalanine metabolism | 8 | 0.104 | ortho-Hydroxyphenylacetic acid | 0.0996 | 1 |
| Taurine and hypotaurine metabolism | 8 | 0.104 | Taurine | 0.0996 | 1 |
| Ascorbate and aldarate metabolism | 9 | 0.117 | myo-Inositol | 0.111 | 1 |
| Vitamin B6 metabolism | 9 | 0.117 | 4-Pyridoxic acid | 0.111 | 1 |
| Nicotinate and nicotinamide metabolism | 15 | 0.195 | Niacinamide | 0.179 | 1 |
| Glycerolipid metabolism | 16 | 0.208 | Glycerol | 0.19 | 1 |
| Fructose and mannose metabolism | 20 | 0.26 | Sorbitol | 0.231 | 1 |
| Pantothenate and CoA biosynthesis | 20 | 0.26 | Ureidopropionic acid | 0.231 | 1 |
| Alanine, aspartate, and glutamate metabolism | 28 | 0.364 | N-Acetylaspartylglutamic acid | 0.309 | 1 |
| Glutathione metabolism | 28 | 0.364 | Spermidine | 0.309 | 1 |
| Inositol phosphate metabolism | 30 | 0.39 | myo-Inositol | 0.327 | 1 |
| Sphingolipid metabolism | 32 | 0.416 | Sphinganine | 0.345 | 1 |
| Arginine and proline metabolism | 36 | 0.468 | Spermidine | 0.379 | 1 |
| Biosynthesis of unsaturated fatty acids | 36 | 0.468 | Stearic acid | 0.379 | 1 |
| Fatty acid degradation | 39 | 0.507 | Palmitoylcarnitine | 0.403 | 1 |
| Tyrosine metabolism | 42 | 0.546 | Homovanillic acid | 0.427 | 1 |
| Amino sugar and nucleotide sugar metabolism | 42 | 0.546 | N-Acetylgalactosamine | 0.427 | 1 |
| Primary bile acid biosynthesis | 46 | 0.598 | Taurine | 0.457 | 1 |

Abbreviation: FDR, false discovery rate
