# Supplementary material for: Metabolic phenotypes of doxorubicin-induced cardiotoxicity among patients with breast cancer
Source: Metabolomics. 2026 Jul 4;22(4):119. doi: 10.1007/s11306-026-02469-7 (PMC13332971; doi:10.1007/s11306-026-02469-7)
Supplement: Supplementary file 2 — Supplementary Material 2 [file 11306_2026_2469_MOESM2_ESM.docx]

**SUPPLEMENTARY METHODS**

**Sample preparation**

All plasma samples were stored at −80°C until processed. On the extraction day, frozen samples were thawed on ice. Samples were prepared with the automated MicroLab STAR system from Hamilton Company. A volume of 100 µL of each sample was transferred into a well of a deep-well plate. Before extraction, several recovery standards were added for quality-control purposes. To remove protein, dissociate small molecules bound to protein or trapped in the precipitated protein matrix, and recover chemically diverse metabolites, proteins were precipitated with 500 µL of methanol under vigorous shaking for 2 minutes (Glen Mills Geno Grinder 2000) followed by a 10-minute centrifugation at 680 g. The resulting extract was divided into 5 fractions: 2 for analysis by 2 separate reverse phase (RP)/UPLC-MS/MS methods with positive ion mode electrospray ionization (ESI), 1 for analysis by RP/UPLC-MS/MS with negative ion mode ESI, 1 for analysis by HILIC/UPLC-MS/MS with negative ion mode ESI, and 1 sample was reserved for backup. Samples were placed briefly on a TurboVap (Zymark) to remove the organic solvent. The sample extracts were stored overnight under nitrogen before preparation for analysis. The untargeted metabolomic analysis was performed at Metabolon, Inc.

**Quality assurance and quality control (QA/QC)**

Several types of controls were analyzed in concert with the experimental samples: a quality-control sample generated by taking a small volume of each experimental sample to serve as a technical replicate throughout the dataset; extracted water samples served as process blanks; a mixture of quality-control standards, carefully chosen to not interfere with the measurement of endogenous compounds, were spiked into every analyzed sample, which allowed us to monitor instrument performance and aided in chromatographic alignment. Instrument variability was determined by calculating the median relative standard deviation for the standards that were added to each sample prior to injection into the mass spectrometers. Overall, process variability was determined by calculating the median relative standard deviation for all endogenous metabolites (i.e., non-instrument standards) present in 100% of the pooled matrix samples. To avoid correlation between sample classes and injection order LC-MS analysis samples were randomized with quality-control samples spaced evenly among the injections.

**Ultra-high-performance liquid chromatography-tandem mass spectroscopy**

All methods used a Waters ACQUITY ultra-performance liquid chromatography (UPLC) and a Thermo Scientific Q-Exactive high-resolution/accurate mass spectrometer interfaced with a heated electrospray ionization (HESI-II) source and Orbitrap mass analyzer operated at 35,000 mass resolution. The sample extract was dried then reconstituted in solvents compatible with each of the 4 methods. Each reconstitution solvent contained a series of standards at fixed concentrations to ensure injection and chromatographic consistency. One aliquot was analyzed with acidic positive ion conditions chromatographically optimized for more hydrophilic compounds. In this method, the extract was gradient eluted from a C18 column (Waters UPLC BEH C18-2.1x100 mm,1.7 mm) with water and methanol containing 0.05% perfluoropentanoic acid (PFPA) and 0.1% formic acid (FA). Another aliquot was analyzed with acidic positive ion conditions; however, it was chromatographically optimized for more hydrophobic compounds. In this method, the extract was gradient eluted from the same as before mentioned C18 column with methanol, acetonitrile, water, 0.05% PFPA, and 0.01%FA and was operated at an overall higher organic content. Another aliquot was analyzed with basic negative ion optimized conditions on a separate dedicated C18 column. The basic extracts were gradient eluted from the column with methanol and water, however with 6.5mM Ammonium Bicarbonate at pH 8. The fourth aliquot was analyzed via negative ionization following elution from a HILIC column (WatersUPLCBEHAmide2.1x150mm,1.7mm) with a gradient consisting of water and acetonitrile with 10mM Ammonium Formate, pH 10.8. The MS analysis alternated between MS and data-dependent MSn scans with dynamic exclusion. The scan range varied slighted between methods but covered 70–1000 m/z. Raw data files were archived and processed as mentioned in method section. All MS2 spectra were collected with a data-dependent acquisition method.
